# “Buen vivir es estar en paz y armonía con todo lo que nos rodea”: estudio cualitativo sobre el buen vivir en el pueblo indígena Kankuamo de Colombia

**DOI:** 10.1590/0102-311XES190223

**Published:** 2024-08-26

**Authors:** Catharina van der Boor, Giovanna Catalina Sánchez-Díaz, Luisa Juliana Guevara-Morales, Carlos Iván Molina-Bulla, Diana Marcela Agudelo-Ortiz, Adolfo José Montero-Villazón, Mario de Jesús Villazón-Rodríguez, Lilibeth Maestre-Arias, Diego Mauricio Aponte-Canencio

**Affiliations:** 1 London School of Hygiene and Tropical Medicine, London, U.K.; 2 Universidad Externado de Colombia, Bogotá, Colombia.; 3 Kankuama IPS, Valledupar, Colombia.; 4 Comisión de Salud del Pueblo Kankuamo, Valledupar, Colombia.

**Keywords:** Pueblos Indígenas, Salud Mental, Autodeterminación, Calidad de Vida, Indigenous Peoples, Mental Health, Self Determination, Quality of Life, Povos Indígenas, Saúde Mental, Autodeterminação, Qualidade de Vida

## Abstract

El pueblo Kankuamo es uno de los 102 pueblos originarios de Colombia, que se encuentran en el proceso de desarrollo de estrategias para la autogestión de salud individual y colectiva. Este artículo tiene como objetivo investigar, identificar y analizar, de forma colaborativa e intercultural, los factores que influyen en el bienestar del pueblo Kankuamo, utilizando el Enfoque de Capacidades propuesto por Amartya Sen. Con este fin, se llevaron a cabo tres grupos focales con la participación de 37 personas de las 15 comunidades del resguardo Kankuamo. Las transcripciones fueron analizadas mediante un análisis temático. De los grupos focales surgieron tres temas centrales para el bienestar de los Kankuamo: (i) armonía entre la naturaleza y los seres humanos, (ii) convivencia social y (iii) autodeterminación. Estos temas reflejan los principios y valores fundamentales que guían a la comunidad hacia el bienestar, la expansión de sus capacidades, la armonía y el desarrollo integral. Los resultados sugieren que los aspectos materiales desempeñan un papel secundario en el concepto de bienestar de la comunidad Kankuamo, y además confirman que es fundamental considerar una visión colectiva de capacidades, no solo individuales, en contextos indígenas. Estudios como este pueden contribuir al desarrollo de enfoques más contextualmente apropiados para evaluar y medir la calidad de vida y el bienestar de las comunidades indígenas, incluyendo el pueblo Kankuamo.

## Abstract (en)

The Kankuamo people are one of the 102 native peoples of Colombia, who are developing strategies for individual and collective health self-management. This article aims to investigate, identify and analyze, collaboratively and interculturally, the factors that influence the well-being of the Kankuamo people, using the Capabilities Approach proposed by Amartya Sen. To this end, three focus groups were conducted with the participation of 37 people from the 15 communities of the Kankuamo reservation. The transcripts were analyzed by means of a thematic analysis. Three central themes for the well-being of the Kankuamo emerged from the focus groups: (i) harmony between nature and human beings, (ii) social coexistence and (iii) self-determination. These themes reflect the fundamental principles and values that guide the community towards a good life, expansion of their capabilities, harmony and holistic development. The results suggest that material aspects play a secondary role in the Kankuamo community’s concept of good life, and confirm that it is fundamental to consider a collective vision of capabilities, not only individual ones, in indigenous contexts. Studies such as this one can contribute to the development of more contextually appropriate approaches to assess and measure the quality of life and well-being of Indigenous communities, including the Kankuamo people.

## Resumo

O povo Kankuamo é um dos 102 povos indígenas da Colômbia que estão em processo de desenvolvimento de estratégias para a autogestão da saúde individual e coletiva. Este artigo tem como objetivo investigar, identificar e analisar, de forma colaborativa e intercultural, os fatores que influenciam o bem-estar do povo Kankuamo, usando a Abordagem de Capacidades proposta por Amartya Sen. Para isso, foram realizados três grupos focais com a participação de 37 pessoas das 15 comunidades da reserva Kankuamo. Análise temática foi usada nas transcrições. Três temas centrais para o bem-estar dos Kankuamo emergiram dos grupos focais: (i) harmonia entre a natureza e os seres humanos, (ii) coexistência social e (iii) autodeterminação. Esses temas refletem os princípios e valores fundamentais que orientam a comunidade para o bem-estar, a expansão de suas capacidades, a harmonia e o desenvolvimento integral. Os resultados sugerem que os aspectos materiais desempenham um papel secundário no conceito de bem-estar da comunidade Kankuamo e confirmam ainda que é essencial considerar uma visão coletiva das capacidades, e não apenas individual, em contextos indígenas. Estudos como este podem contribuir para o desenvolvimento de abordagens mais adequadas ao contexto para avaliar e medir a qualidade de vida e o bem-estar das comunidades indígenas, incluindo o povo Kankuamo.

## Introducción

### Pueblos indígenas en Colombia

En Colombia y América Latina, las comunidades étnicas enfrentan condiciones materiales y sociales desiguales que las colocan en una situación socioeconómica más vulnerable en comparación con la población no racializada. Estas condiciones se traducen en peores indicadores de salud, como un acceso limitado a los servicios médicos, mayor mortalidad, falta de aseguramiento sanitario, deficiencias en las condiciones alimentarias y menores probabilidades de supervivencia ante enfermedades ^1^ ^,^ ^2^ ^,^ ^3^ ^,^ ^4^ ^,^ ^5^.

Esto los hace más propensos a sufrir altas tasas de depresión, suicidio, abuso de sustancias y otros trastornos mentales ^6^ ^,^ ^7^ ^,^ ^8^ ^,^ ^9^ ^,^ ^10^ ^,^ ^11^ ^,^ ^12^ ^,^ ^13^, afectando su calidad de vida, dignidad y su capacidad para ejercer sus libertades fundamentales desde sus cosmovisiones y prácticas socioculturales ^9^ ^,^ ^10^ ^,^ ^12^.

A pesar de las leyes promulgadas para respaldar su participación en el Sistema General de Seguridad Social y proteger sus derechos, como el Decreto-Ley 4.633 de 2011 ^14^ para medidas de asistencia y reparación, el Decreto 1.973 de 2013 ^15^ que crea la Subcomisión de Salud Indígena, y el Decreto 1.953 de 2014 ^16^ que establece un régimen especial para la administración de sistemas propios, como el Sistema Indígena de Salud Propia e Intercultural (SISPI), persisten desafíos en la implementación efectiva de estas políticas.

El nuevo orden constitucional de Colombia reconoce al país como una nación pluriétnica y multicultural, con obligación estatal de proteger y respetar esta diversidad. Sin embargo, el acceso a servicios de salud adecuados sigue siendo un desafío para muchos grupos indígenas, cuya concepción de salud es integral y está intrínsecamente ligada a su relación con el mundo y el medioambiente. A pesar de los avances legislativos, como el *Decreto 1.811 de 1990*, la *Ley 21 de 1991* y los decretos reglamentarios, se necesita un enfoque más concertado que reconozca y respete las particularidades culturales y étnicas de estos pueblos en el ámbito de la salud.

La descentralización administrativa y la figura del resguardo indígena ofrecen oportunidades para la creación de un sistema de salud indígena que responda a las necesidades reales de los pueblos indígenas.

Estas políticas, en particular el SISPI, plantean inquietudes sobre la generación de indicadores de salud y bienestar que trascienden la perspectiva epidemiológica biomédica, considerando las cosmovisiones propias de los pueblos. Para muchos de estos pueblos, la salud se entiende como el “buen vivir”, que abarca aspectos físicos, mentales, sociales, culturales, espirituales y territoriales, arraigados en su cosmovisión. Esto fomenta un diálogo constante entre saberes ancestrales y prácticas cotidianas, como el fortalecimiento de la cultura, la soberanía alimentaria, la medicina tradicional y la relación con la naturaleza y el territorio.

Para abordar de manera efectiva los factores fundamentales para la salud y el buen vivir en estos pueblos, es esencial comprender primero qué significa una buena vida para ellos. El objetivo principal de este artículo es investigar y analizar colaborativamente el significado del buen vivir para la comunidad indígena Kankuamo de Colombia, usando el Enfoque de Capacidades.

### El Enfoque de Capacidades

El Enfoque de Capacidades de Sen ^17^ analiza el bienestar considerando las circunstancias de cada sociedad y busca promover el desarrollo humano y la justicia social. Destaca el fortalecimiento de las libertades, la capacidad de acción y la ampliación de oportunidades y habilidades individuales y comunitarias para una vida autónoma y en consonancia con los valores ^17^ ^,^ ^18^. En este enfoque, “capacidades” se refiere a las posibilidades de las personas para llevar la vida que valoran, mientras que “funcionamientos” se refiere a lo que finalmente logran ser o hacer ^19^. La calidad de vida y el bienestar se basan en las capacidades de las personas y las comunidades para lograr funcionamientos significativos, sin limitarse a ingresos o recursos y sin reducirse al placer ^17^. Sen subraya la importancia de la autonomía personal y social, incluyendo el derecho a una cultura e identidad propia que dé sentido a la vida y permita una existencia plena y valiosa ^20^ ^,^ ^21^.

Dentro del Enfoque de Capacidades, es esencial dar prioridad a la perspectiva única de cada comunidad sobre el “vivir bien” al abordar intervenciones para promover la salud y el bienestar ^22^. Este enfoque permite rescatar el desarrollo de capacidades desde las comunidades indígenas, incorporando sus saberes y conocimientos ancestrales. En este artículo, se presentan los resultados de la investigación realizada con el Pueblo Kankuamo, en la cual se analizan las capacidades que influyen en su concepto de buen vivir.

### El pueblo Kankuamo

A diferencia de sus vecinos Iku, Kogui y Wiwa, la historia del pueblo Kankuamo ha transitado por un sendero distinto, marcado por la necesidad de restaurar lazos que unen a sus miembros y reconstruir su identidad colectiva. Esta compleja trayectoria se enraíza en dos fenómenos interconectados que han impactado profundamente en su memoria y en su cultura: la colonización y el conflicto armado. La colonización europea dejó una huella profunda en el pueblo Kankuamo, sembrando la idea de que su extinción era inevitable. Durante el siglo XX, generaciones vivieron fracturadas en su identidad étnica, bajo el peso de un relato nacional que privilegiaba el mestizaje y el blanqueamiento. Solo la Constitución Política de 1991 marcó un giro hacia el reconocimiento de la diversidad cultural, abriendo la puerta a la reconstrucción identitaria.

Durante el conflicto armado en Colombia, la violencia contra el pueblo Kankuamo y otros pueblos indígenas se manifestó en varios niveles y fue perpetrada por distintos actores como las guerrillas, grupos armados paramilitares, el ejército, grupos o bandas criminales y la delincuencia común. Además, se involucraron grandes empresas y compañías multinacionales extractivas que se beneficiaron de la desigual tenencia de la tierra, expropiada a las comunidades indígenas, causando desplazamientos masivos.

El conflicto armado devastó el territorio Kankuamo, dejando un legado de dolor y desarraigo. La desaparición forzada y el desplazamiento masivo fragmentaron las comunidades y arrebataron a muchos de su tierra natal. Las nuevas generaciones han crecido bajo la sombra de estas atrocidades, enfrentando un profundo desarraigo cultural. Esto ha dado como resultado el desplazamiento forzado del 40% de su población, la ocupación de sus territorios por diversos actores, y la persecución, desaparición y asesinato de sus líderes sociales y espirituales, mamos y representantes Kankuamos ^23^ ^,^ ^24^.

A pesar de estas adversidades, el pueblo Kankuamo ha demostrado una extraordinaria capacidad de resiliencia. En la actualidad, se encuentran en un proceso de revitalización cultural conocido como el “Renacer Kankuamo” o “El Proceso”, luchando por restaurar su memoria colectiva y reconstruir su identidad como pueblo.

La conexión con los pueblos hermanos de la Sierra Nevada y la posibilidad de reencontrarse en la Ley de Origen ^25^, la cual es un conjunto de normas y principios ancestrales que guían su cosmovisión, prácticas culturales y organización social, ha sido fundamental. En esencia, el “Renacer” representa un resurgimiento cultural al significar la revitalización de las tradiciones, valores y prácticas ancestrales. Un camino hacia la autonomía por el fortalecimiento del autogobierno y la libre determinación. Un reencuentro con el ser, sentir y pensar Kankuamo al fortalecer la identidad Kankuama como base para el desarrollo cultural y social, y un lazo con los miembros de la comunidad y de los otros pueblos hermanos alrededor de la Ley de Origen ^25^.

El “Renacer Kankuamo” se erige en un faro que ilumina el camino hacia la preservación de la identidad cultural del pueblo Kankuamo. Esta iniciativa, impulsada por las Autoridades del Cabildo, la Organización Indígena Kankuama y el Congreso del Pueblo Kankuamo, como máxima autoridad, representa un esfuerzo colectivo por salvaguardar los valores ancestrales y fortalecer la autonomía del pueblo. En este contexto, Kankuama IPS se suma como una pieza fundamental en este proceso de revitalización cultural. Esta entidad prestadora de salud, profundamente arraigada en los principios de la Ley de Origen, no solo vela por el bienestar físico y mental de la comunidad Kankuamo, sino que también se convierte en un bastión para la recuperación y el fomento de las prácticas y saberes tradicionales en salud.

Kankuama IPS va más allá de la atención médica convencional, reconociendo la profunda conexión que existe entre la salud y la cosmovisión del pueblo Kankuamo. Por ello, integra a su modelo de atención elementos propios de la medicina tradicional, respetando la sabiduría ancestral y promoviendo su revitalización entre las nuevas generaciones. Dentro de sus objetivos, se encuentra garantizar el derecho a la salud integral y al buen vivir por medio de la atención médica en el contexto del SISPI; para esto, trabaja en sintonía con las iniciativas políticas y organizativas adelantadas por la comunidad para conocer las necesidades y requerimientos de sus habitantes.

El propósito de este artículo es ofrecer una base de conocimiento, desde una perspectiva comunitaria e intercultural, sobre los factores más relevantes para entender el buen vivir en el pueblo Kankuamo a través de grupos focales participativos. Esto se hace con el objetivo de fortalecer al proceso político-organizativo del pueblo Kankuamo para la autonomía y la gobernanza en salud.

## Metodología

### Contexto del estudio

Considerando el contexto histórico, detallado anteriormente, y en consonancia con los objetivos planteados en el estudio, se optó por una metodología cualitativa, de diálogo de saberes en el marco de un proyecto más amplio de recuperación del conocimiento ancestral, de un perfil epidemiológico y aproximación al bienestar kankuamo. Esta metodología permitió captar los significados, sentires y experiencias colectivas construidas en la comunidad en torno al concepto del “buen vivir” o “vivir bien”. A su vez, se mostró respetuosa con el proceso político-organizativo de fortalecimiento y recuperación de la memoria Kankuama. Para la recolección de datos, se emplearon grupos focales como técnica de investigación que permite la interpretación de actitudes, conocimientos, reacciones y experiencias frente a un tema en común dentro de un contexto grupal.

Los grupos focales se llevaron a cabo en el marco del tercer encuentro de saberes *Lógicas de Bienestar: Salud Mental, Vivir y Convivir*, un evento de dos días realizado en la comunidad de Los Háticos. Este encuentro, organizado por la Universidad Externado de Colombia, la Universidad de Liverpool (Reino Unido), en colaboración con la Kankuama IPS y el Cabildo Indígena Kankuamo, contó con la participación activa de miembros de la comunidad Kankuama, incluyendo médicos tradicionales, sabedores, autoridades en salud, jóvenes y estudiantes. El objetivo principal del encuentro fue reflexionar sobre las formas de convivencia que impactan el bienestar comunitario y contribuyen a la gobernanza. A través de diálogos enriquecedores, los participantes exploraron las lógicas de bienestar propias del pueblo Kankuamo, identificaron recursos y capacidades para fortalecer el buen vivir, la convivencia y la construcción de paz.

Este encuentro de saberes formó parte de un entramado de diálogos desarrollados en cinco fases o encuentros, enmarcados en la metodología de Diálogo de Saberes. Esta metodología, promovida por la Universidad Externado de Colombia, se erige en un puente fundamental para construir una salud intercultural que mejore el acceso a los servicios de salud, especialmente en el ámbito de la atención primaria en salud, así como permitió una articulación favorable con el Enfoque de Capacidades aportado por la Universidad de Liverpool.

### Diseño del estudio

Para cumplir con los objetivos planteados se realizaron tres grupos focales.

### Participantes y reclutamiento

Participaron 27 mujeres y 10 hombres de 13 de las 15 comunidades del resguardo Kankuamo (Ramalito, Atánquez, Rancho de la Goya, Murillo, Valledupar, Guatapurí, Chemesquemena, La Mina, Pontón, Las Flores, Laureles, Los Háticos y Mojao; Río Seco y Pueblo Bello no tuvieron participantes), seleccionados mediante muestreo intencional durante el encuentro de saberes. Se organizaron en tres grupos focales basados en la proximidad y el número de participantes de cada comunidad (Tabla 1). Los criterios de participación incluyeron ser miembro de la comunidad Kankuama y mayor de 18 años. Los grupos focales tuvieron una duración promedio de 1 hora y 45 minutos.

**Tabla 1:** Organización de los grupos focales y características demográficas de los participantes.

Grupo focal	Duración	Participantes	Comunidades	Mujeres	Hombres
1	1 hora y 45 minutos	13	Guatapurí	3	1
			Chemesquemena	6	0
			Los Háticos	1	2
2	1 hora y 43 minutos	12	Atánquez	4	0
			Pontón	1	0
			Las Flores	1	0
			Valledupar	3	0
			Laureles	0	3
3	1 hora y 42 minutos	12	Ramalito	3	1
			La Mina	1	1
			Mojao	1	1
			Murillo	0	1
			Rancho de la Goya	3	0

El proyecto contó con la aprobación ética otorgada por el Comité de Ética de la Universidad Externado de Colombia (número de referencia: concepto nº 27). Todos los participantes dieron su consentimiento verbal para unirse a los grupos focales, habiendo otorgado previamente su consentimiento por escrito para el encuentro de saberes, lo cual incluye el consentimiento de publicación de datos anonimizados.

### Procedimiento

Se llevaron a cabo los grupos focales el 3 de octubre de 2021 en Los Háticos, una de las 15 comunidades del Resguardo Indígena Kankuamo. Cuatro investigadores de la Universidad Externado de Colombia, junto con un representante del pueblo Kankuamo (Diana Marcela Agudelo-Ortiz, Carlos Iván Molina-Bulla, Diego Mauricio Aponte-Canencio, Luisa Juliana Guevara-Morales y Adolfo José Montero-Villazón), previamente capacitados en el Enfoque de Capacidades ^17^, realizaron los grupos focales. Al inicio de cada sesión, se comunicaron los objetivos de la actividad y se proporcionaron aclaraciones éticas sobre la confidencialidad y el manejo de datos. Todos los grupos se grabaron en audio.

Después del consentimiento, los participantes se presentaron especificando su comunidad. Se utilizó una guía de entrevista semiestructurada con cinco preguntas abiertas (Cuadro 1) para facilitar los grupos focales, permitiendo a los participantes reflexionar sobre sus propias capacidades, siguiendo la conceptualización de Sen ^26^.

**Cuadro 1:** Guía de entrevista semiestructurada.

GUÍA DE ENTREVISTA SEMIESTRUCTURADA
¿Qué entiende cada uno por el “buen vivir” o “el vivir bien”? ¿Cuáles son los aspectos o elementos valiosos que contribuyen al “buen vivir” o al “vivir bien”? ¿Cuáles son los aspectos o elementos que afectan o amenaza el “buen vivir” o al “vivir bien”? ¿Qué acciones toma la comunidad para proteger y contribuir al “buen vivir” o el “vivir bien”? ¿Qué oportunidades y libertades valora cada quien en su vida y en la vida de la comunidad para garantizar un “buen vivir” o un “vivir bien”?

Al finalizar cada grupos focales, se brindó un espacio para que cada participante compartiera brevemente sus impresiones sobre cómo se sintió durante la actividad, los conocimientos adquiridos y sus contribuciones finales.

### Análisis de datos

Las grabaciones de audio fueron transcriptas textualmente y anonimizadas por las investigadoras (Luisa Juliana Guevara-Morales y Giovanna Catalina Sánchez-Díaz) con el apoyo de los enlaces territoriales (Almis Montero-Villazon y Angela Cordoba). Las transcripciones fueron analizadas por la autora C. van der Boor usando un análisis temático, el cual se hizo siguiendo las seis fases propuestas por Braun & Clarke ^27^. El análisis, realizado en NVivo (https://lumivero.com/products/nvivo/), fue revisado por el equipo de investigación y por tres representantes del pueblo Kankuamo conjuntamente.

## Resultados

**Figura 1:**
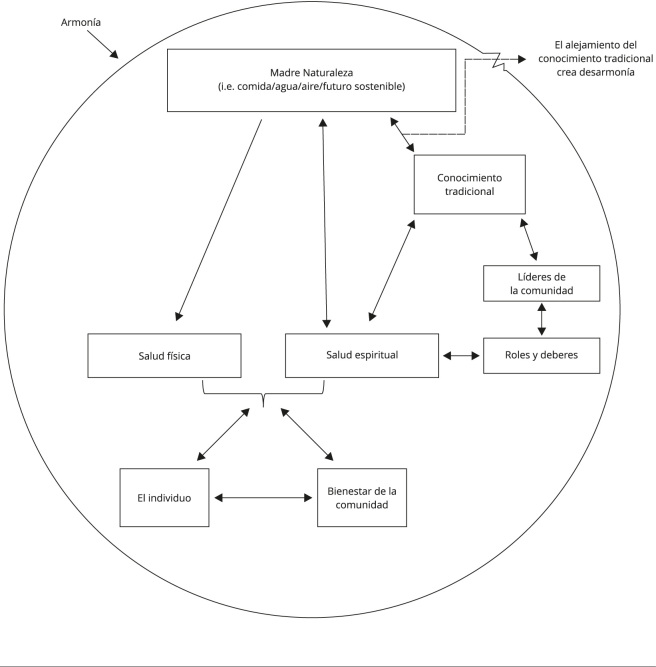
Ley de Origen Kankuamo.

El análisis de los resultados de los grupos focales partió por la comprensión de la cosmología y el concepto de “ser, sentir y pensar Kankuamo” desde la perspectiva de la Ley de Origen. Con base en esto, se creó un mapa conceptual (Figura 1) para visualizar la relación entre estos conceptos.

La Ley de Origen se define como la relación de armonía y equilibrio entre todos los seres, personas, naturaleza y espíritus que gobiernan la vida dentro del pueblo Kankuamo. Esta ley establece las normas y costumbres dentro de la comunidad destacando el profundo lazo que tienen con su territorio ancestral, considerándolo fuente de vida, identidad cultural, y espiritualidad. Constituye la narrativa que da sentido, pervivencia y pertenencia al pueblo Kankuamo; en ella reside la explicación de su origen, tejiendo un vínculo indisoluble entre su historia, su cosmovisión y su propósito colectivo.

“*Para mí el vivir bien significa estar bien espiritualmente, cumplir con los mandatos de La Ley de Origen*” (grupo focal 3, hombre).

**Figura 2:**
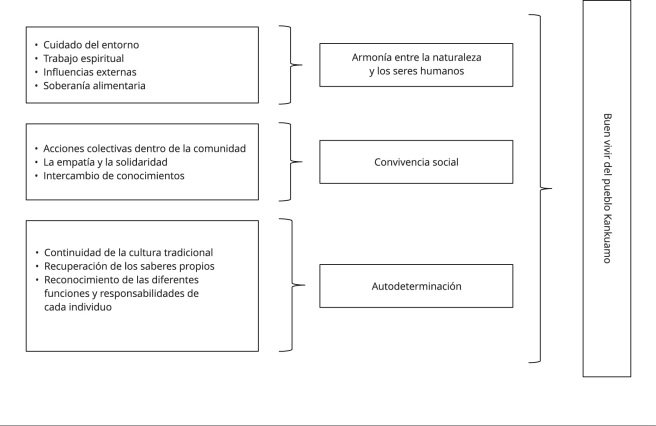
Temas de análisis de los tres grupos focales.

En los tres grupos focales, surgieron tres temas centrales para el buen vivir Kankuamo: (i) la armonía entre la naturaleza y los seres humanos, (ii) la convivencia social y (iii) la autodeterminación (Figura 2). Estos temas reflejan los principios y valores que guían a la comunidad hacia la expansión de sus capacidades individuales y colectivas. La armonía dentro de la Ley de Origen permite a los individuos tener las libertades de ser y hacer lo que valoran como parte del pueblo Kankuamo.

### Armonía entre la naturaleza y los seres humanos: “para mí el buen vivir es estar bien con la naturaleza”

Los tres grupos focales revelan la importancia de la armonía entre la naturaleza y los humanos para el buen vivir Kankuamo. Esta armonía se fomenta a través de la espiritualidad, el equilibrio, la relación con la naturaleza, el bienestar integral y la salud holística, involucrando al individuo, la familia, la comunidad y el entorno natural.

El cuidado del entorno, incluyendo la Sierra Nevada de Santa Marta, considerada el “Corazón del Mundo” por los Kankuamo, es crucial para preservar esta armonía. La comunidad asume la responsabilidad sagrada de protegerla mediante la prevención de la contaminación, el impacto externo y la soberanía alimentaria.

Sin embargo, la comunidad enfrenta desafíos para preservar este equilibrio, como el impacto persistente del conflicto armado. La pérdida de miembros, el desplazamiento, la persecución y la estigmatización debilitan a los principios ancestrales y a las capacidades individuales y colectivas, amenazando la expansión de capacidades y la sostenibilidad del buen vivir.

“*Pues hablando sobre las amenazas que de pronto nos afecta el vivir bien es pensar que tal vez vuelva el conflicto armado porque cuando vino eso con las bombas que hacían, las minas que colocaban afectaban a la naturaleza, afectaban sitios sagrados, afectaban a los árboles y también los animales terminaban con esos, entonces eso también nos afectaba el buen vivir*” (grupo focal 3, mujer).

Además del conflicto armado, se abordó el impacto negativo de influencias externas en la comunidad, como la minería, la deforestación y la contaminación, que afectan el equilibrio y la armonía del territorio, tanto física como espiritualmente, generando preocupaciones sobre el futuro.

“*Nuestros mayores nos dicen que si nosotros no cuidamos y protegemos el río y no protegemos el territorio, vamos a llegar, nos va a llegar el caso que tenemos que ir a comprar la botellita de agua para podernos bañar*” (grupo focal 1, mujer).

Se reconoce la capacidad de autosuficiencia, pero el turismo externo afecta la seguridad y el acceso a lugares sagrados.

“*Nosotros tenemos todo en la Sierra para vivir sin necesidad de lo que venga de afuera, ¿qué hay que hacer? Fortalecerlo, cuidarlo, ni acabar con la cultura, con los trabajos tradicionales, con lo espiritual que tenemos en la Sierra. Y pues segundo, todo lo que viene de afuera es dañino, es con todo lo que llega a la Sierra con la intención de hacer explotaciones se basa solamente en el signo pesos*” (grupo focal 3, hombre).

Las influencias externas dañan la naturaleza y la dimensión espiritual del territorio, requiriendo trabajo espiritual para restaurar el equilibrio. La salud espiritual es vital para la armonía, y su afectación exige acciones de limpieza y protección por parte de las autoridades Kankuamas.

La compra de alimentos externos preocupa por la soberanía alimentaria y su impacto en la salud física. Recuperar la autonomía alimentaria es crucial para evitar riesgos de enfermedades, destacándola como capacidad fundamental para la comunidad Kankuama.

“*Para nosotros también el buen vivires comer bien, comer bien porque si nosotros comemos bien comemos cosas propias, cosas de nosotros, cosas que cultivamos acá, siempre vamos a estar bien saludable no nos vamos a enfermar*” (grupo focal 1, mujer).

El COVID-19 ha motivado el retorno al cultivo propio a través del confinamiento. Esto promueve el intercambio, el trueque, la autonomía alimentaria y el cuidado de la comunidad.

“*Algo que nosotros pudimos notar, ahora, digamos en el marco de la pandemia fue precisamente ese uso y aprovechamiento de la tierra incluyendo pues el tema de la alimentación fue muy notable*” (grupo focal 2, mujer).

El acceso a los cultivos propios se basa en la relación bidireccional entre la naturaleza y los seres humanos.

“*Estar bien significa proteger esos bienes y servicios que nosotros tomamos de la Madre, significa también el retribuir a la Madre lo que nosotros tomamos de ella*” (grupo focal 3, hombre).

### Convivencia social: “el buen vivir es también como el saber convivir con los demás, el tratar bien a nuestro vecino, a nuestro amigo, al de afuera”

La comunidad Kankuama logra el buen vivir a través de relaciones sociales que incluyen roles comunitarios, respeto ambiental y autoridades culturales, promoviendo la solidaridad y la participación. En los grupos focales, se enfatizaron las acciones colectivas basadas en la empatía, la solidaridad y el conocimiento compartido para una vida valiosa.

“*Yo pienso que para mí el buen vivir es tener buena convivencia, no tanto tener un buen trabajo porque la plata va y viene, si no tener buena comunicación con nuestros vecinos, buena convivencia, compartir lo poco que tengo con el otro, para mí eso es buen vivir*” (grupo focal 2, mujer).

Las acciones colectivas se basan en el apoyo mutuo para mejorar la vida en comunidad. La guardia indígena vela por la seguridad del territorio, mientras que las labores comunitarias, como la reparación de vías y el apoyo a desplazados, fomentan la tranquilidad y el bienestar colectivo.

“*Cuando sucede una calamidad familiar, nosotros nos unimos y hacemos recolecta, campañas económicas, o puede ser también de ropa, de alimento, de para hacerle llegar a esa persona que le esté sucediendo algo, es como, ahí usamos como eso, la empatía*” (grupo focal 3, hombre).

Estos valores se basan en la disposición y comunicación adecuadas, así como en las relaciones de reciprocidad y unidad entre los diferentes miembros.

“*Una manera de durar y trabajar en forma colectiva, de pronto algo que le llamamos ‘manovuelta’. Por ejemplo, si usted necesita limpiar su finca... nos ponemos de acuerdo los campesinos, y todos vamos a esa parte, eso es, como prestándole el día, y así sucesivamente nos vamos rotando cuando usted necesite ese día, se lo devolvemos, y esa es una manera de tratar, de ayudarnos unos al otro*” (grupo focal 3, hombre).

El intercambio de conocimiento tradicional, liderado por los mayores, promueve la coexistencia pacífica y la expansión de capacidades. Este conocimiento se refuerza en el hogar y en las actividades tradicionales, asegurando la permanencia de saberes propios con roles distintos para los mayores y las familias.

“*Yo voy a las kankuruas ya porque me nace de corazón, porque eso que voy a recibir ahí en ese espacio me va a servir a mí, ya después ahí si van a fluir esas energías positivas* (...) *he ido reflejando eso en mi hija*” (grupo focal 1, mujer).

### Autodeterminación: “construyendo con las demás comunidades en unidad para ir apropiándose de lo propio”

La comunidad Kankuama basa su desarrollo de capacidades en la autodeterminación. Rescatando saberes ancestrales, han reconstruido normas y costumbres para enfrentar influencias externas. La principal amenaza es la vulneración de sus derechos como indígenas.

Los participantes enfatizaron sobre la importancia de preservar su cultura, recuperar saberes y reconocer roles individuales para alcanzar el buen vivir anhelado.

La continuidad de lo tradicional se ve desafiada por influencias externas que afectan su autonomía. Un ejemplo es la tecnología, que impacta en la gobernanza Kankuamo.

“*Nuestro territorio se ha venido afectando también con la tecnología ¿por qué? Porque ese es un elemento que ha enfermado como a los jóvenes como a los adultos* (...) *cuando ya llega uno acá a estos espacios todo mundo con el teléfono prendido*” (grupo focal 2, hombre).

El pueblo Kankuamo trabaja en la recuperación y preservación de sus saberes ancestrales afectados por episodios de violencia e invasiones históricas, desde la conquista hasta el conflicto armado. Esto comienza con la reafirmación del territorio propio: “*como indígenas el buen vivir es tener su territorio, tener territorio porque si nosotros no tenemos territorio tenemos inestabilidad*” (grupo focal 2, mujer).

Se destacó la urgencia de restaurar y preservar saberes ancestrales como el tejido de mochilas, la medicina tradicional y los espacios culturales. Los mayores y las autoridades, guardianes de este conocimiento, lo transmiten a las nuevas generaciones. Sin embargo, la pandemia de COVID-19 amenaza con su pérdida.

“*Algo fundamental que ha sido importante para salvaguardar ese buen vivir, es la forma como se ha obtenido el conocimiento de esos mayores, de esas bibliotecas vivas.* (...) *esa extracción o recopilación del conocimiento de nuestros mayores y mayoras para la apropiación de uno y retribuirla de generación en generación*” (grupo focal 2, mujer).

La reconstrucción debe ser un proceso conjunto entre las comunidades; “*para que sea un buen vivir tenemos que irla compartiendo y armando y enseñando las comunidades que tienen el poquito de saber*” (grupo focal 2, hombre). Esto se logra a través de espacios para difundir conocimientos y acciones concretas como comisiones, grupos de danza y música tradicional, sirviendo como modelo para promover el bienestar individual y colectivo.

Cada persona tiene una función y responsabilidad dentro de la comunidad: “*hay una libertad, y esa libertad pues lógicamente, se da también, bajo el parámetro de unos deberes* (...)*, los deberes que tenemos nosotros como miembros de una etnia, es que hay que cumplir lo que está establecido en la ley de origen*” (grupo focal 3, hombre).

Dentro de la ley de origen se reconoce el rol tradicional de la mujer; “*la mayoría de las mujeres digamos territorialmente muchas mujeres contribuimos con elementos de nuestro cuerpo para esos sitios tradicionales*” (grupo focal 2, mujer). A su vez, se evidencia un cambio en los roles de género, con las mujeres ganando gradualmente espacios en sus comunidades, y equilibrando los sistemas de gobernanza y comités. Así, las mujeres han adquirido nuevas capacidades a lo largo de estos procesos (grupo focal 2):

Mujer participante: “*y también posicionar a las mujeres al tema de niveles políticos porque es que aquí no se reconocía a las mujeres en espacios políticos*”.

Entrevistador: “*¿ese es un elemento que ha contribuido?*”.

Mujer participante: “*si claro, nosotros hoy en día tenemos voz y voto*”.

Mujer participante 2: “*sí, son acciones que van más allá de la parte tradicional, porque para mantener ese equilibrio no solo vamos a hacer trabajos tradicionales sino también acciones visibles*”.

## Discusión

Este artículo tuvo como objetivo investigar los factores que son importantes para el buen vivir del pueblo indígena Kankuamo, usando la propuesta teórica del Enfoque de Capacidades ^17^. Aunque varios estudios previos han explorado este concepto en pueblos indígenas de Latinoamérica ^28^ ^,^ ^29^, solo algunos ^30^ ^,^ ^31^ ^,^ ^32^ ^,^ ^33^ lo han hecho usando este marco teórico usando datos primarios. Por tal motivo, previamente se realizó una exhaustiva revisión focal o de alcance sobre investigaciones relacionadas con el Enfoque de Capacidades, el buen vivir y los conocimientos y saberes propios en salud en comunidades indígenas ^34^.

Del análisis cualitativo surgieron tres temas centrales: la armonía entre la naturaleza y los seres humanos, la convivencia social y la autodeterminación. Estos temas centrales son fundamentales para la expansión de capacidades dentro del pueblo Kankuamo, permitiendo funcionamientos individuales y colectivos valorados por ellos mismos. Esto sugiere que los aspectos materiales desempeñan un papel secundario en su concepción del buen vivir. En cambio, valores como el conocimiento tradicional, prácticas espirituales, valores compartidos entre comunidades y la visión de futuro son los pilares centrales para el desarrollo de capacidades y el buen vivir. Este estudio evidencia la importancia de determinar local y participativamente las capacidades relevantes para diferentes personas y contextos.

La armonía entre la naturaleza y los seres humanos es esencial para el pueblo Kankuamo, basada en la Ley de Origen que explica la interrelación de todas las cosas desde el inicio del universo. Ellos ven la naturaleza como espiritual y no separan lo social de lo natural, son interdependientes. A diferencia del mundo Occidental, no la ven como un recurso material o económico, sino como crucial para preservar su identidad cultural y bienestar colectivo.

El Enfoque de Capacidades valora la naturaleza por su contribución al bienestar y la agencia individual de dos maneras ^19^ ^,^ ^35^. En primer lugar, el valor del mundo natural se reconoce cuando refleja la agencia. Sen sugiere que debemos considerar diferentes formas de valorar el mundo natural, lo que requiere un diálogo intercultural para comprender estos valores en diversos contextos ^21^. En el caso de los Kankuamo, estos valores se basan en la Ley de Origen, que enfatiza la importancia de la agencia tanto individual como colectiva. Además, los Kankuamo consideran como de su responsabilidad cuidar el entorno y que el entorno cuide de ellos, lo que implica una obligación mutua en lugar de depender exclusivamente de la agencia humana. Esto subraya la importancia de adoptar una perspectiva más colectiva de las capacidades en contextos indígenas, respaldada por hallazgos previos en otros pueblos indígenas ^33^ ^,^ ^36^ ^,^ ^37^.

En segundo lugar, el Enfoque de Capacidades dicta preservar y expandir las libertades y capacidades de las personas sin comprometer las de las generaciones futuras ^5^. Esto coincide con las preocupaciones de los Kankuamo sobre la explotación desmedida y contaminación de recursos naturales que desplaza a comunidades y restringe su acceso a tierras, agua y plantas medicinales. Además, la dependencia de alimentos externos preocupa por su impacto negativo en la salud, afectando así a futuras generaciones también.

La convivencia social es fundamental para el buen vivir Kankuamo, definido colectivamente a través de una sólida identidad comunitaria y visión del mundo. Las capacidades sociales involucran acciones colectivas, empatía, solidaridad y el intercambio de conocimientos, manteniendo la armonía según la Ley de Origen. Al igual que en otras comunidades indígenas, la cooperación y la reciprocidad son vitales para la vida y el bienestar ^29^ ^,^ ^33^ ^,^ ^37^. La tradición oral Kankuamo promueve el compartir el conocimiento como un acto colectivo que amplía las libertades del pueblo, como se ha visto en otras comunidades indígenas ^20^ ^,^ ^38^ ^,^ ^39^. El buen vivir no se limita al bienestar individual, sino que es inseparable del bienestar comunitario.

El tema de la autodeterminación surge a través de la continuidad cultural, la recuperación de saberes propios y el reconocimiento de diferentes roles en la comunidad. En las Naciones Unidas se reconoce el derecho de los pueblos indígenas a determinar su estatus político y buscar su desarrollo ^40^. Sen enfatiza la autodeterminación política en el Enfoque de Capacidades, incluyendo la participación en decisiones políticas ^19^ ^,^ ^26^. Sin embargo, los Kankuamo enfrentan limitaciones sistemáticas debido a la historia de la conquista, el colonialismo y el conflicto armado, como discriminación, abandono estatal, violación de derechos humanos y falta de reconocimiento cultural. Aunque la autodeterminación es central, la narrativa subraya las barreras persistentes.

La recuperación de saberes propios y la autodeterminación fortalecen la comunidad Kankuama. La pandemia de COVID-19 ha desafiado esta recuperación, pero ha impulsado el retorno de familias al territorio, revitalizado así prácticas ancestrales. Para lograrlo, es esencial tomar decisiones centradas en la comunidad y enriquecer su bienestar en medio de los cambios por la pandemia. Los gobiernos y organizaciones internacionales deben apoyar la expansión de capacidades y la autodeterminación de las poblaciones indígenas ^41^. Esto fortalecerá la resiliencia del pueblo Kankuamo ante futuros desafíos, permitiéndoles buscar su buen vivir de manera autónoma o en diálogo constructivo con instancias externas.

### Fortalezas y limitaciones

Al finalizar cada grupo focal, se proporcionó un espacio para que los participantes compartieran sus impresiones. Valoraron expresarse libremente y ser considerados expertos, lo que empoderó la discusión. Se enfatizó la importancia de identificar y fortalecer capacidades individuales y colectivas, y de escuchar las opiniones de los demás para promover el buen vivir en la comunidad.

Los hallazgos cualitativos de este estudio, junto con investigaciones adicionales realizadas a lo largo de tres años, contribuyeron al desarrollo de una *Escala de Bienestar Kankuamo* diseñada para medir la salud mental y el bienestar del pueblo Kankuamo (artículo bajo revisión).

Por otro lado, es importante reconocer que todos los participantes de los grupos focales están involucrados en iniciativas para recuperar y fortalecer los saberes Kankuamo, lo que podría limitar la generalización de los resultados a toda la comunidad. También existen desafíos en capturar la profundidad de los relatos debido a posibles diferencias en perspectivas entre los participantes y los investigadores.

## Conclusión

El estudio actual fue el primero en utilizar el Enfoque de Capacidades para explorar los determinantes del buen vivir para el pueblo Kankuamo. Los tres temas centrales de armonía entre la naturaleza y los seres humanos, convivencia social y autodeterminación se destacaron como determinantes del buen vivir, y los participantes discutieron los facilitadores y barreras que experimentan actualmente en cada uno de estos ámbitos. Estos tres temas brindan un punto de partida útil para determinar políticas, programas e intervenciones que puedan ayudar a promover el bienestar y la expansión de las capacidades colectivas e individuales más ampliamente del pueblo Kankuamo. Además, se destacan la importancia de utilizar enfoques de investigación participativos. Los hallazgos cualitativos sirvieron para la construcción del instrumento *Escala de Bienestar Kankuamo* diseñado para la medición de la salud mental y el bienestar del pueblo Kankuamo desde un Enfoque de Capacidades. En general, esta investigación destaca las posibilidades y los beneficios de operacionalizar el Enfoque de Capacidades para comprender el bienestar a partir de las condiciones particulares que presenta el pueblo Kankuamo.
